# Taxonomy and phylogeny of *Litschauerella* (*Hymenochaetales*) and *Tubulicium* (*Trechisporales*) in East Asia: Three new species and six new records

**DOI:** 10.3897/mycokeys.138.203229

**Published:** 2026-08-13

**Authors:** Yi-Chung Lin, Che-Chih Chen

**Affiliations:** 1 Department of Biology, National Museum of Natural Science, Taichung 404023, Taiwan Department of Biology, National Museum of Natural Science Taichung Taiwan; 2 Department of Plant Pathology, National Chung Hsing University, Taichung 402202, Taiwan Department of Plant Pathology, National Chung Hsing University Taichung Taiwan

**Keywords:** China, corticioid fungi, environmental sequence annotation, *
Hydnodontaceae
*, *
Hymenochaetales
*, *
Litschauerellaceae
*, Taiwan, *
Trechisporales
*, Vietnam

## Abstract

*Litschauerella* and *Tubulicium* are corticioid genera that share distinctive lyocystidia with multiple roots but differ in the chemical reactions and surface ornamentation of these structures, as well as in basidiospore morphology. Their familial placements have long remained unresolved due to a lack of molecular data for the type species. Here, we present the first nuc rDNA internal transcribed spacer ITS1-5.8S-ITS2 (ITS) sequence for *Litschauerella abietis*, the type species of *Litschauerella*, and integrate it with nuc 28S rDNA (28S) sequences and morphological re-examination of type materials and new collections from East Asia to infer the phylogenetic placements of both genera. Phylogenetic analyses based on combined ITS and 28S datasets using Bayesian inference and maximum likelihood methods confirm that *Litschauerella* forms a monophyletic, well-supported clade within *Litschauerellaceae* of the *Hymenochaetales*, representing an early-diverging lineage within the order, whereas *Tubulicium* is retained within *Hydnodontaceae* of the *Trechisporales*, sister to *Membranohymenium*. Based on morphological and molecular evidence, three new species of *Tubulicium* are described from East Asia: *T. densum***sp. nov**., *T. fusiforme***sp. nov**., and *T. naviculisporum***sp. nov**. Additionally, five species are newly recorded for Taiwan (*L. abietis*, *T. bambusicola*, *T. curvisporum*, *T. dussii*, and *T. vermiferum*), and *L. hastata* is reported as a new record for Vietnam. Updated identification keys are provided for both genera.

## Introduction

*Litschauerella* Oberw. and *Tubulicium* Oberw. are small, poorly known corticioid genera of wood-inhabiting fungi. They were both established by Oberwinkler (1966), with *L. abietis* (Bourdot & Galzin) Oberw. and *T. vermiferum* (Bourdot) Oberw. designated as type species. These genera share several morphological traits, including resupinate, thin basidiomata with a hispidulous hymenial surface, a monomitic hyphal system with nodose-septate hyphae, conical to cylindrical lyocystidia with multiple roots, and subclavate basidia with slight constrictions (Oberwinkler 1966). However, *Litschauerella* is distinguished by its lyocystidia encrusted with irregular, pyramidal crystals, fibrous hyphae, dextrinoid reactions in the inner layer, and broad ellipsoid to nearly globose basidiospores, often with inconspicuous ornamentation (Kisimova-Horovitz et al. 1997a). By contrast, *Tubulicium* has lyocystidia covered with dendroidly branching hyphae, a weakly amyloid inner layer, and navicular to vermicular basidiospores (Kisimova-Horovitz et al. 1997a; Bernicchia and Gorjón 2010).

Currently, the genus *Litschauerella* comprises four species: *L. abietis*, *L. clematidis* (Bourdot & Galzin) J. Erikss. & Ryvarden, *L. gladiola* (G. Cunn.) Stalpers & P.K. Buchanan, and *L. hastata* (G. Cunn.) Stalpers & P.K. Buchanan (Eriksson and Ryvarden 1976; Jülich 1979; Stalpers and Buchanan 1991). The genus *Tubulicium* includes eleven species: *T. bambusicola* S.H. He & S.L. Liu, *T. curvisporum* Ushijima & N. Maek., *T. dussii* (Pat.) Oberw. ex Jülich, *T. erectum* Hjortstam & Ryvarden, *T. filicicola* (G. Cunn.) Oberw., *T. junci-acuti* Boidin & Gaignon, *T. papillatosporum* G. Moreno & Esteve-Rav., *T. ramonense* Kisim.-Hor., Oberw. & L.D. Gómez, *T. raphidisporum* (Boidin & Gilles) Oberw., Kisim.-Hor. & L.D. Gómez, *T. vermiferum*, and *T. yunnanense* S.Yuan He & C.L. Zhao (Oberwinkler 1966; Jülich 1979; Boidin and Gaignon 1992; Kisimova-Horovitz et al. 1997a, 1997b; Moreno 2007; Hjortstam and Ryvarden 2008; Liu et al. 2019b; Ushijima et al. 2019; Wijesinghe et al. 2025). In East Asia, *T. bambusicola*, *T. curvisporum*, *T. raphidisporum*, *T. vermiferum* and *T. yunnanense* have been recorded (Maekawa 1992; Wu 2000; Wu 2002; Wu and Chen 2002; Liu et al. 2019b; Ushijima et al. 2019; Maekawa 2021; Wijesinghe et al. 2025), whereas only *L. clematidis* and *L. gladiola* have so far been reported (Maekawa 1993; Liu et al. 2019b; Maekawa 2021).

At the higher taxonomic level, Larsson et al. (2004) showed that *T. vermiferum*, the type species of *Tubulicium*, belongs to the trechisporoid clade together with *Porpomyces mucidus* (Pers.) Jülich, *Sistotremastrum niveocremeum* (Höhn. & Litsch.) J. Erikss., *Subulicystidium* spp., and *Trechispora* spp. Subsequently, Larsson (2007) placed *Tubulicium* in *Hydnodontaceae* within the *Trechisporales*, a placement further supported by subsequent phylogenetic studies within the order (Liu et al. 2019b, 2022). With morphological affinities to *Tubulicium*, *Litschauerella* was also regarded as a possible member of *Hydnodontaceae*, though without molecular confirmation (Larsson 2007; Hibbett et al. 2014). Recently, Liu et al. (2019b) generated the first ITS and 28S sequences of *L. gladiola*, and Liu et al. (2022) placed it in the *Hymenochaetales* rather than *Trechisporales*. Although *L. gladiola* is not the type species, it further questioned the placement of the genus in the *Trechisporales*. Consequently, *Litschauerella* was temporarily excluded from the *Trechisporales* (Liu et al. 2022). However, its higher-level placement and generic circumscription remain unresolved, as the type species *L. abietis* has not yet been sequenced.

Here, we reassess the phylogenetic placement and generic limits of *Litschauerella* and *Tubulicium* in East Asia by integrating ITS and 28S sequence data with detailed morphological re-examination of type materials and new collections. We describe and illustrate newly discovered species, clarify their phylogenetic affinities, and provide updated identification keys for both genera.

## Materials and methods

### Morphological studies

The studied specimens were deposited at the fungaria of the National Museum of Natural Science, Taiwan (TNM), the New Zealand Fungarium, New Zealand (PDD), and the Swedish Museum of Natural History, Sweden (S). Acronyms follow the Index Herbariorum (http://sweetgum.nybg.org). Basidiomata were photographed using an Olympus TG-4 or a Canon EOS R digital camera. The hymenial surface was examined under an Olympus SZ51 (Olympus, Tokyo, Japan) stereomicroscope at magnifications up to × 50. Free-hand thin sections of basidiomata were mounted in 5% potassium hydroxide (KOH) with 1% phloxine for observation and measurement, and in Melzer’s reagent to test for amyloidity and dextrinoidity, using a ZEISS Axioscope 5 microscope (Carl Zeiss, Germany) at magnifications up to × 1000. Microscopic measurements were performed using the digital image analysis software ImageJ v. 1.54r, calibrated against a stage micrometer at each magnification used. The following abbreviations are used: L = mean basidiospore length (arithmetic average of all basidiospores), W = mean basidiospore width (arithmetic average of all basidiospores), Q = L/W ratio, n = number of basidiospores measured per specimen, m = meters above sea level.

### DNA extraction, polymerase chain reaction (PCR), and DNA sequencing

DNA was extracted from either dried specimens or mycelia cultivated on 2% MEA. Tissue disruption and homogenization were performed prior to extraction using liquid nitrogen and TissueLyser II (QIAGEN, Hilden, Germany). DNA was extracted using the DNeasy Plant Mini Kit (QIAGEN, Hilden, Germany) or the NautiaZ Plant DNA Extraction Mini Kit (Nautia Gene, Taipei, Taiwan) following the manufacturers’ protocols.

The study targeted two genetic regions: the nuc rDNA internal transcribed spacer ITS1-5.8S-ITS2 (ITS) region was amplified using the primer pair ITS1F/ITS4B (Gardes and Bruns 1993), while the nuc 28S rDNA (28S) was amplified using primer pair LR0R/LR5 (Vilgalys and Hester 1990). For aged specimens, the ITS region was amplified in two segments using the primer pairs ITS1/fITS7R (ITS1 and partial 5.8S) and 58A1F/ITS4 (partial 5.8S and ITS2) (White et al. 1990; Martin and Rygiewicz 2005; Tedersoo et al. 2017). Missing data at sequence junctions were denoted with the ambiguity code “n”. Amplifications were conducted in a Bio-Rad T100™ Thermal Cycler (Bio-Rad, Hercules, California, USA). The PCR conditions for all regions were as follows: initial denaturation at 94 °C for 5 min, followed by 36 cycles of 94 °C for 30 s, 55 °C for 30 s, and 72 °C for 1 min, and a final extension at 72 °C for 7 min. PCR products were purified and sequenced using the Sanger method by Genomics BioSci & Tech (New Taipei, Taiwan). Newly generated sequences were manually assembled using BioEdit v. 7.2.5 (Hall 1999) or Geneious Prime 2024.0.5 (https://www.geneious.com) and submitted to GenBank (https://www.ncbi.nlm.nih.gov/genbank/). Accession numbers are listed in Table 1. Consensus sequence accuracy and identity were verified by comparison with BLAST results from GenBank entries.

**Table 1.** Species and sequences used in the phylogenetic analyses. Newly generated sequences are indicated in bold, and sequences derived from holotype specimens are marked with an asterisk (*).

**Scientific name**	**Specimen no / Isolation**	**ITS**	**28S**	**Location**	**References**
*Allotrechispora daweishanensis*	CLZhao 17860*	MW302337	MW293866	China	Zong et al. (2021)
*A. gatesiae*	LWZ 20180515-18*	OM523378	OM339206	Australia	Liu et al. (2022)
*A. xantha*	CLZhao 2632*	MW302339	MW293868	China	Zong et al. (2021)
*Auricularia* sp.	PBM2295	DQ200918	AY634277	USA	Unpublished
*Brevicellicium exile*	5217MD	HE963777	HE963778	Spain	Telleria et al. (2013)
*B. olivascens*	KHL 8571	HE963792	HE963793	Sweden	Telleria et al. (2013)
*Dextrinocystis calamicola*	He 5693	MK204533	MK204546	China	Liu et al. (2019b)
*D. calamicola*	He 5701	MK204534	MK204547	China	Liu et al. (2019b)
*Echinoporia hydnophora*	LWZ 20150802-9	ON063639	ON063838	China	Wang et al. (2023)
*Exidiopsis calcea*	MW 331	AF291280	AF291326	Germany	Weiß and Oberwinkler (2001)
*Fasciodontia brasiliensis*	MSK-F 7245a	MK575201	MK598734	Brazil	Yurchenko et al. (2020)
*F. yunnanensis*	LWZ 20190811-50a	ON063675	ON427360	China	Wang et al. (2023)
*Fibrodontia alba*	EYu110703-25*	KC928274	KC928275	Taiwan	Yurchenko and Wu (2014)
*F. brevidens*	Wu 9807-16	KC928276	KC928277	Vietnam	Yurchenko and Wu (2014)
*F. gossypina*	AFTOL 599	DQ249274	AY646100	Unknown	Unpublished
*Fomitopsis pinicola*	AFTOL 770	AY854083	AY684164	Unknown	Lutzoni et al. (2004)
*Fulvoderma australe*	LWZ 20190809-39b	ON063644	ON063843	China	Wang et al. (2023)
*Fuscoporia sinica*	LWZ 20190816-19a	ON063649	ON427358	China	Wang et al. (2023)
*Grandicium tenuissimum*	CLZhao 37275	PX108623	PV975048	China	Wijesinghe et al. (2025)
*G. tenuissimum*	CLZhao 37540*	PX108624	PV975049	China	Wijesinghe et al. (2025)
*Grifola frondosa*	AFTOL 701	AY854084	AY629318	Unknown	Lutzoni et al. (2004)
*Hymenochaete colliculosa*	Dai 16429	MF370597	–	China	He et al. (2017)
*H. xerantica*	LWZ 20190814-13b	ON063657	ON063856	China	Wang et al. (2023)
*Hyphodontia borbonica*	FR-0219441	KR349240	–	France: Réunion	Riebesehl et al. (2015)
*H. pachyspora*	LWZ 20170908-5*	MT319426	MT319160	China	Wang et al. (2021)
*H. pallidula*	Kotiranta 18839	OP620785	OP620785	Finland	Viner et al. (2023)
*H. zhixiangii*	LWZ 20170818-13	MT319420	MT319151	China	Wang et al. (2021)
*Inonotus hispidus*	LWZ 20180703-1	ON063659	ON063858	China	Wang et al. (2023)
*Kneiffiella eucalypticola*	LWZ 20180509-11	MT319410	MT319142	China	Wang et al. (2021)
*K. floccosa*	Berglund 150-02	DQ873618	DQ873618	Sweden	Larsson et al. (2006)
*K. subglobosa*	LWZ 20180416-6	MT319413	MT319145	China	Wang et al. (2021)
*Leifia brevispora*	LWZ 20170820-48	MK343470	MK343474	China	Liu et al. (2019a)
*L. flabelliradiata*	KG Nilsson 36270	DQ873635	DQ873635	Sweden	Larsson et al. (2006)
***Litschauerella abietis***	**Chen 2742**	**PX060163**	–	**Taiwan**	**Present study**
*L. gladiola*	He 3171	MK204555	MK204556	China	Liu et al. (2019b)
*Luellia cystidiata*	JHP-09.455	MW371211	MW371211	Denmark	Larsson et al. (2004)
*Lyomyces crustosus*	LWZ 20170815-23	MT319465	MT319201	China	Wang et al. (2021)
*L. leptocystidiatus*	LWZ 20170814-14*	MT319429	MT319163	China	Wang et al. (2021)
*L. sambuci*	LWZ 20180905-1	MT319444	MT319178	China	Wang et al. (2021)
“*Membranohymenium grandinioides*”	CLZhao 37657	PX108625	–	China	Wijesinghe et al. (2025)
“*M. grandinioides*”	CLZhao 37768	PX108626	–	China	Wijesinghe et al. (2025)
*Odonticium romellii*	KHL s.n.	DQ873639	DQ873639	Norway	Larsson et al. (2006)
*Palliocystidium chlamydatum*	GG-GUY18-115*	OQ555356	OQ555358	France	Ordynets and Gruhn (2025)
*P. chlamydatum*	GG-GUY18-371	OQ555357	–	France	Ordynets and Gruhn (2025)
*Peniophorella praetermissa*	LWZ 20180903-14	ON063686	ON063886	China	Wang et al. (2023)
*P. pubera*	LWZ 20210624-16b	ON063687	ON063887	China	Wang et al. (2023)
*P. rude*	LWZ 20171026-7	ON063688	ON063888	China	Wang et al. (2023)
*Porpomyces mucidus*	Dai 12692	KT157833	KT157838	Czech Republic	Wu and Zhao (2015)
*P. submucidus*	Cui 5183	KT152143	KT152145	China	Wu and Zhao (2015)
*Pteridomyces galzinii*	Bernicchia 8122	MN937559	MN937559	Italy	Spirin et al. (2021)
*P. galzinii*	GB0150230	LR694210	LR694188	Spain	Unpublished
*Repetobasidium conicum*	KHL 12338	DQ873647	DQ873647	USA	Larsson et al. (2006)
*R. mirificum*	FP-133558-sp	–	AY293208	USA	Binder et al. (2005)
*Resinicium austroasianum*	LWZ 20191208-11	ON063691	ON063891	China	Wang et al. (2023)
*R. bicolor*	AFTOL 810	DQ218310	AY586709	USA	Larsson et al. (2004)
*R. friabile*	LWZ 20210923-23a	ON063692	ON427362	China	Wang et al. (2023)
*Rigidoporus cirratus*	LWZ 20170818-16	ON427472	ON427355	China	Wang et al. (2023)
*R. populinus*	LWZ 20190811-39a	ON063674	ON063874	China	Wang et al. (2023)
*Schizocorticium lenis*	LWZ 20180922-61	ON063698	ON063898	China	Wang et al. (2023)
*S. magnosporum*	Wu 1510-34	MK405351	MK405337	China	Wu et al. (2021)
*S. mediosporum*	LWZ 20180921-7	ON063696	ON063896	China	Wang et al. (2023)
*Sertulicium granuliferum*	He 3338	MK204540	MK204552	China	Liu et al. (2019b)
*S. jacksonii*	Spirin 10425	MN987943	MN987943	Russia	Spirin et al. (2021)
*S. niveocremeum*	KHL13727	MN937563	MN937563	France	Spirin et al. (2021)
*Sidera minutipora*	Cui 16720	MN621349	MN621348	Australia	Du et al. (2020)
*S. srilankensis*	Dai 19654	MN621344	MN621346	Sri Lanka	Du et al. (2020)
*Sistotremastrum suecicum*	KHL 11849	MN937571	MN937571	Brazil	Spirin et al. (2021)
*S. vigilans*	Fonneland 2011-78*	MN937572	MN937572	Norway	Spirin et al. (2021)
*Skvortzovia dabieshanensis*	LWZ 20210918-15b	ON063694	ON063894	China	Wang et al. (2023)
*S. pinicola*	LWZ 20210623-18b	ON063695	ON063895	China	Wang et al. (2023)
*S. qilianensis*	LWZ 20180904-20	ON063693	ON063893	China	Wang et al. (2023)
*Subulicystidium brachysporum*	KHL 16100	MH000599	MH000599	Brazil	Ordynets et al. (2018)
*S. longisporum*	KHL 14229	MH000601	MH000601	Sweden	Ordynets et al. (2018)
*S. nikau*	L 1296	MH041513	MH041565	France: Réunion	Ordynets et al. (2018)
*S. parvisporum*	L 0140*	MH041529	MH041590	France: Réunion	Ordynets et al. (2018)
*S. perlongisporum*	LY 11631*	MN207054	MN207030	France: Réunion	Ordynets et al. (2020)
*S. tropicum*	He 3583*	MK204530	MK204542	China	Liu et al. (2019b)
*Suillosporium cystidiatum*	VS3830	MN937573	MN937573	Russia	Spirin et al. (2021)
*Thelephora ganbajun*	ZRL20151295	LT716082	KY418908	China	Zhao et al. (2017)
*Trechispora farinacea*	KHL 8793	AF347089	AF347089	Unknown	Larsson et al. (2004)
*T. hymenocystis*	KHL 8795	AF347090	AF347090	Sweden	Larsson et al. (2004)
*T. nivea*	GB0102694	KU747096	AY586720	Sweden	Larsson et al. (2004); ITS unpublished
*T. regularis*	KHL 10881	AF347087	AF347087	Jamaica	Larsson et al. (2004)
*Tubulicium bambusicola*	He 4058	MK204535	–	Thailand	Liu et al. (2019b)
*T. bambusicola*	He 4776	MK204536	MK204551	China	Liu et al. (2019b)
***T. bambusicola***	**WEI 19-403**	**PX060165**	–	**Taiwan**	**Present study**
*T. curvisporum*	TUFC 14538	LC213624	LC336428	Japan	Ushijima et al. (2019)
***T. densum***	WEI 17-045*	**PX060166**	**PX048511**	**Taiwan**	**Present study**
***T. dussii***	**WEI 19-508**	**PX060168**	**PX048510**	**Taiwan**	**Present study**
***Tubulicium fusiforme***	Wu 0107-2*	**PX060164**	–	**China: Yunnan**	**Present study**
***Tubulicium naviculisporum***	WEI 18-615*	**PX060167**	**PX048509**	**Taiwan**	**Present study**
*T. raphidisporum*	He 2851	MK204538	MK204554	China	Liu et al. (2019b)
*T. raphidisporum*	He 3191	OM523534	OM339334	China	Liu et al. (2022)
*T. yunnanense*	CLZhao 37459*	PV475577	PV646273	China	Wijesinghe et al. (2025)
*T. yunnanense*	CLZhao 39116	PV475578	–	China	Wijesinghe et al. (2025)
*Tubulicium* sp.	HFRG_SR240206_1	PP979456	PP979456	UK	Unpublished
*Tubulicium* sp.	LWZ 20180414-5	OM523535	OM339335	Malaysia	Liu et al. (2022)
*T. vermiculare*	GEL 5015	–	AJ406424	Unknown	Langer (2002)
*T. vermiferum*	GB: KHL 8714	–	AY463477	Norway	Larsson et al. (2004)
*T. vermiferum*	K(M):194604	MZ159524	–	United Kingdom	Unpublished
*T. vermiferum*	TUMH 40378	LC213625	–	Japan: Tokyo	Ushijima et al. (2019)
*Tubulicrinis albobadius*	CLZhao 26202*	PQ523361	PQ523364	China	Dai et al. (2025)
*T. calothrix*	LWZ 20210919-1b	ON063704	–	China	Wang et al. (2023)
*T. glebulosus*	LWZ 20180903-13	ON063705	ON063905	China	Wang et al. (2023)
*T. subulatus*	LWZ 20190914-7	ON063706	ON063906	China	Wang et al. (2023)
*Umbellus sinensis*	LWZ 20190615-27	OR242616	OR236212	China	Wang and Zhou (2024)
*U. sinensis*	LWZ 20190615-39	OR242617	OR236213	China	Wang and Zhou (2024)
Uncultured *Basidiomycota* (source: orchid root of *Epidendrum firmum*)	BAS011_R045_R3	JX998759	–	Costa Rica	Kartzinel et al. (2013)
Uncultured *Basidiomycota* (source: orchid root of *Epidendrum firmum*)	BAS017_R045_1	JX998764	–	Costa Rica	Kartzinel et al. (2013)
Uncultured *Basidiomycota* (source: orchid root of *Epidendrum firmum*)	BAS027_R013_R1_H02	JX998747	–	Costa Rica	Kartzinel et al. (2013)
Uncultured *Basidiomycota* (source: orchid root of *Epidendrum firmum*)	BAS030BASR007_2	JX998772	–	Costa Rica	Kartzinel et al. (2013)
Uncultured *Basidiomycota* (source: orchid root of *Epidendrum firmum*)	BAS031BASR007_2	JX998773	–	Costa Rica	Kartzinel et al. (2013)
Uncultured *Basidiomycota* (source: roots of *Allanblackia stuhlmannii*)	Derema II 041	KU195513	–	Tanzania	Alvum-Toll (2013)
Uncultured *Basidiomycota* (source: mycorrhizal root of epiphytic orchid)	OTU73	MH005912	–	China	Xing et al. (2019)
Uncultured fungus (source: healthy plant materials)	MYL7403	LC697296	–	Japan	Unpublished
Uncultured soil fungus (source: *Picea mariana* forest soil organic horizon)	OTU748	KF617710	KF617710	USA: Alaska	Taylor et al. (2014)
Uncultured soil fungus (source: humic horizon soil)	TE13 OTU153	EF434078	EF434078	USA: Alaska	Taylor et al. (2007)
Uncultured soil fungus	NCD LSU otu1068	–	KF566038	USA	Mueller et al. (2014)
Uncultured soil fungus	NCD LSU otu315	–	KF565285	USA	Mueller et al. (2014)
Uncultured soil fungus	NCD LSU otu879	–	KF565849	USA	Mueller et al. (2014)
Uncultured soil fungus	OTU97-33	–	JQ311085	USA	Eichorst and Kuske (2012)
*Xylodon flaviporus*	FR-0249797	MH880201	–	Germany	Wang et al. (2021)
*X. nesporii*	LWZ 20190814-17a	ON063679	ON063879	China	Wang et al. (2023)

### Phylogenetic analyses

Sequences of ITS and 28S were aligned using MAFFT v. 7.409 (Katoh and Standley 2013) with the default algorithm. Poorly aligned positions were removed using ClipKIT (Steenwyk et al. 2025). Two concatenated ITS+28S datasets were constructed and deposited at Figshare (https://doi.org/10.6084/m9.figshare.32568702). The first dataset included *Litschauerella* and representatives of the *Hymenochaetales*, with *Thelephora ganbajun* M. Zang selected as the outgroup following Dai et al. (2025). The second dataset included *Tubulicium* and representatives of the *Trechisporales*, with *Kneiffiella floccosa* (Bourdot & Galzin) Jülich and Stalpers selected as the outgroup following Ordynets and Gruhn (2025). Bayesian inference (BI) and maximum likelihood (ML) analyses were then conducted on both datasets using MrBayes v. 3.2.6 (Ronquist et al. 2012) and RAxML BlackBox (Stamatakis 2014), respectively. Both BI and ML analyses were performed at the CIPRES Science Gateway (Miller et al. 2010).

For the BI analyses, jModelTest v. 2.1.10 (Darriba et al. 2012) was used to estimate the best-fit substitution model for each partition (ITS1, 5.8S, ITS2, and 28S) based on the Akaike information criterion (AIC). The four partitions were assigned independent substitution models and parameters in the BI analyses. Markov chain Monte Carlo (MCMC) searches were run for 10 million generations, with four chains and trees sampled every 1000 generations. The first 25% of trees (2,500) were discarded as burn-in while the remaining trees were used to construct the 50% majority-rule consensus phylogram with posterior probabilities (PP). For the ML analysis, the best-scoring tree with bootstrap (BS) support values was computed under a GTRGAMMA model with 1000 bootstrap replicates, followed by a thorough ML search. Gaps were treated as missing data, and branches were considered well supported when PP ≥ 0.9 and BS ≥ 70%. The phylogenetic trees were visualized and edited in TreeGraph2 (Stöver and Müller 2010) and Adobe Illustrator 27.9 (Adobe Systems, Inc).

## Results

### Molecular phylogeny of *Litschauerella* and *Tubulicium*

**Figure 1.**
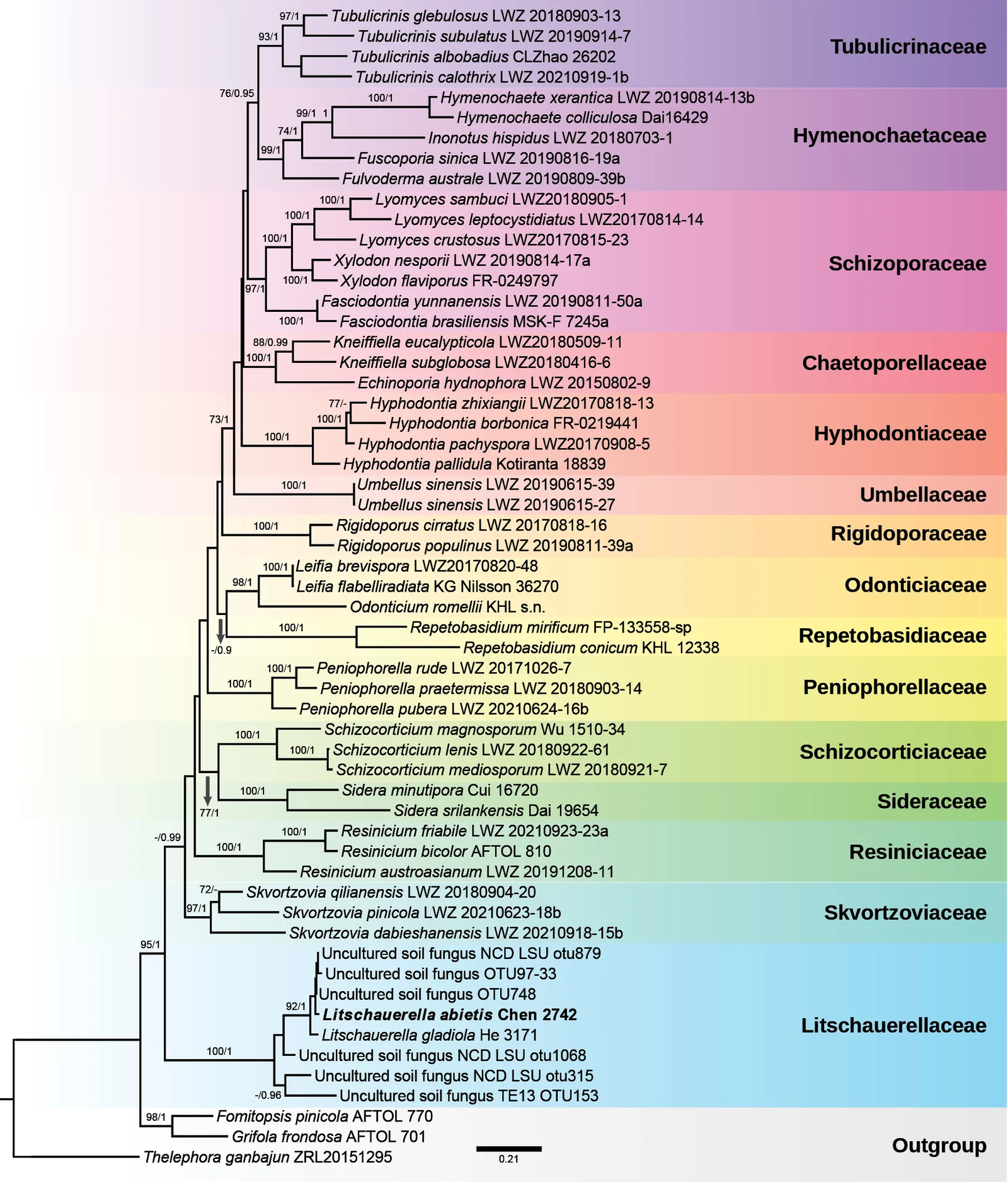
Phylogenetic relationships of *Litschauerellaceae* and related families within the *Hymenochaetales* inferred from combined ITS+28S dataset using ML analysis. The phylogram shows the placement of *Litschauerella* within the family *Litschauerellaceae*. Branch nodes are labelled with ML bootstrap values and Bayesian posterior probabilities. Boldface indicates specimens sequenced in this study.

**Figure 2.**
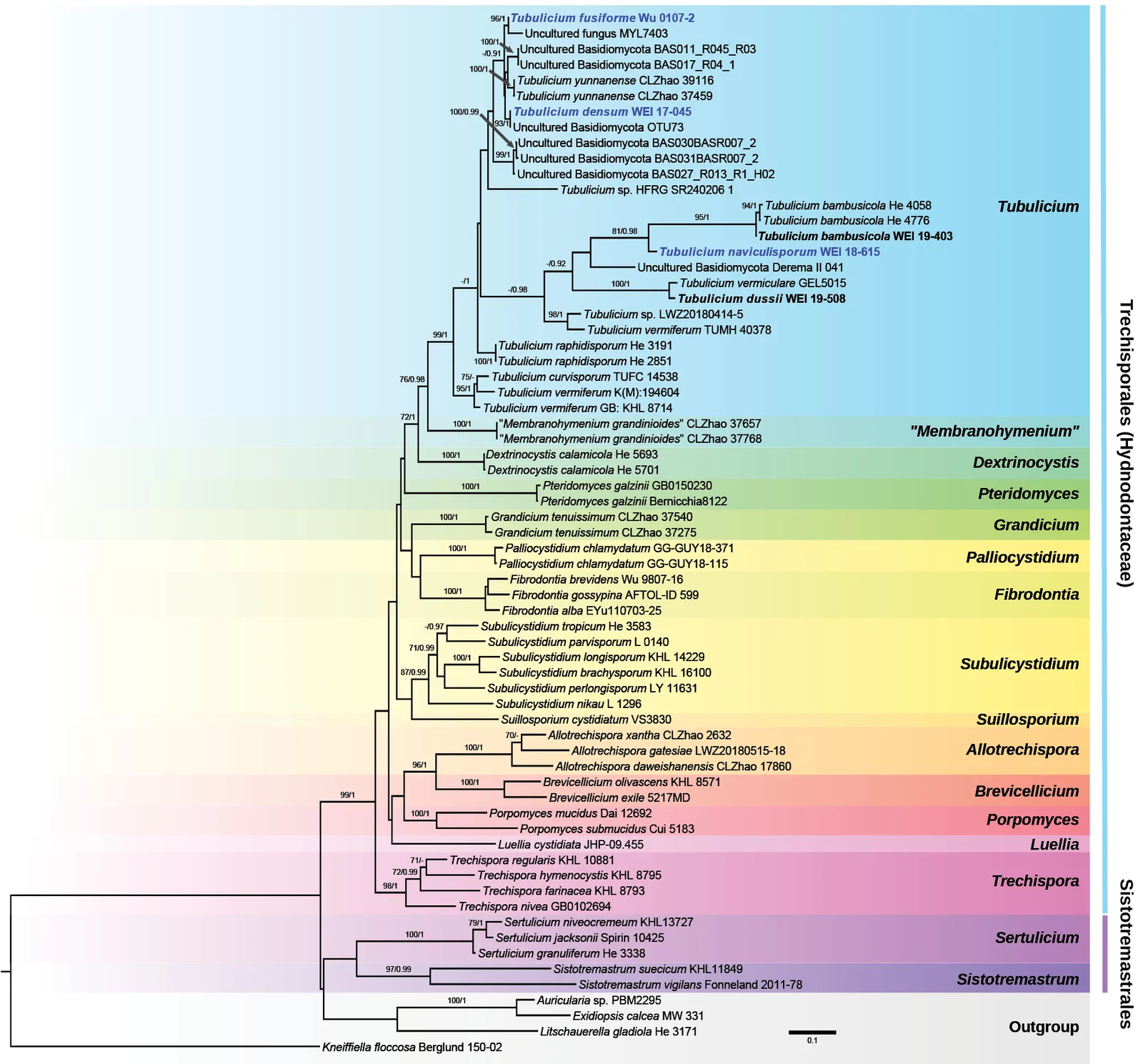
Phylogenetic relationships of *Tubulicium* within *Hydnodontaceae* of the *Trechisporales* inferred from combined ITS+28S dataset using ML analysis. Branches are labelled with ML bootstrap support values and Bayesian posterior probabilities. Blue labels indicate the three new species described in this study, and boldface indicates specimens sequenced in this study.

The concatenated ITS+28S datasets used to infer the phylogenetic relationships of *Litschauerella* within the *Hymenochaetales* and *Tubulicium* within the *Trechisporales* comprised 57 and 67 sequences, respectively. The *Hymenochaetales* alignment comprised 1,611 characters with gaps (753 bp from ITS and 858 bp from 28S), whereas the *Trechisporales* alignment consisted of 1,981 characters with gaps (638 bp from ITS and 1,343 bp from 28S). For both datasets, GTR+I+G was selected as the best-fit substitution model for the ITS1, ITS2, and 28S partitions, whereas K80+I and SYM+I+G were selected for the 5.8S partition in the *Hymenochaetales* and *Trechisporales* datasets, respectively. The topologies inferred from BI and ML analyses were largely congruent; therefore, we present only the ML phylograms (Figs 1, 2), with both bootstrap support (BS) and Bayesian posterior probability (PP) values indicated at the nodes.

In the *Litschauerella* phylogeny (Fig. 1), *L. abietis* (*Chen 2742*), the type species of the genus, together with *L. gladiola* and six OTUs of uncultured fungi, formed a monophyletic and well-supported clade corresponding to *Litschauerella* and *Litschauerellaceae* (BS = 100%, PP = 1). These results confirm the phylogenetic placement of *Litschauerella* and *Litschauerellaceae* within the *Hymenochaetales*, where the clade represents an early-diverging lineage within the order.

In the *Tubulicium* phylogeny (Fig. 2), *T. vermiferum*, the type species of the genus, together with representative species, formed a monophyletic and well-supported clade within the *Trechisporales* (BS = 99%, PP = 1), sister to the genus *Membranohymenium* Ying Xu & C.L. Zhao. Three newly described species (*T. densum*, *T. fusiforme*, and *T. naviculisporum*) each formed distinct, well-supported lineages. *T. densum* (*WEI 17-045*) clustered with an uncultured *Basidiomycota* OTU73 (BS = 93%, PP = 1), while *T. fusiforme* (*Wu 0107-2*) grouped with an uncultured fungus (MYL7403; BS = 96%, PP = 1). *T. naviculisporum* (*WEI 18-615*) was represented by a single sequence and formed a sister lineage to *T. bambusicola*.

The monotypic genus *Membranohymenium*, typified by *M. grandinioides* Ying Xu & C.L. Zhao (Wijesinghe et al. 2025), is indicated by quotation marks in Fig. 2 because the ITS sequence of the holotype specimen (*CLZhao 37769*; GenBank: PX108627) is inconsistent with those of the paratype specimens. The ITS sequence of *CLZhao 37769* shares 99% similarity (676/681 bp) with that of the holotype of *Merulius tomentopileatus* (C.L. Zhao) C.L. Zhao (*CLZhao 9563*; GenBank: MT020743), a species of *Meruliaceae (Polyporales)*. This result suggests that PX108627 may not represent *M. grandinioides*. Therefore, PX108627 was excluded from our phylogenetic analyses. The two paratype specimens of *M. grandinioides* (*CLZhao 37657* and *CLZhao 37768*) formed a well-supported monophyletic lineage (BS = 100%, PP = 1; Fig. 2).

## Taxonomy

### *Hymenochaetales* Oberw., in Frey, Hurka & Oberwinkler, Beitr. Biol. Pfl.: 89. 1977

#### Litschauerellaceae

Taxon classificationFungiHymenochaetalesLitschauerellaceae

Jülich, Bibl. Mycol. 85:377. 1982

B15F7F4B-655E-5CEE-8B19-260E502B2B0C

##### Notes.

*Litschauerellaceae* is a monotypic family with *Litschauerella* as its type genus. Jülich (1982) simultaneously established *Litschauerellaceae* (typified by *Litschauerella*) and *Hydnodontaceae* (typified by *Hydnodon* Banker). Most subsequent treatments adopted the latter name and regarded *Litschauerellaceae* as its synonym (Larsson 2007; Hjortstam and Ryvarden 2008; Bernicchia and Gorjón 2010). Based on phylogenetic analyses presented in this study (Fig. 1), we recognize *Litschauerellaceae* as an independent family within the *Hymenochaetales*, distinct from *Hydnodontaceae* in the *Trechisporales*.

#### Litschauerella

Taxon classificationFungiHymenochaetalesLitschauerellaceae

Oberw., Sydowia 19:43. 1966

BCE7C04D-C78D-50EF-8863-2B5D9D127375

##### Notes.

Four species are currently recognized in *Litschauerella*: *L. abietis*, *L. clematidis*, *L. gladiola*, and *L. hastata*. Members of the genus are characterized by resupinate basidiomata, a monomitic hyphal system with nodose-septate hyphae, and lyocystidia densely encrusted with minute crystals. The basidiospores are globose to subglobose, often with inconspicuous ornamentation, except in *L. hastata*, which bears subglobose to broadly ellipsoid basidiospores. The present study confirms the monophyly of *Litschauerella* and supports its placement in the family *Litschauerellaceae* of the *Hymenochaetales* (Fig. 1).

#### Litschauerella abietis

Taxon classificationFungiHymenochaetalesLitschauerellaceae

(Bourdot & Galzin) Oberw. ex Jülich, Persoonia 10: 335. 1979

98202085-C597-51FC-ADA3-CEC53CED2C9B

Figs 3A, 3B, 4

≡ Peniophora aegerita subsp. abietis Bourdot & Galzin, Bull. Soc. Mycol. France 28:383. 1913.

##### Descriptions and illustrations.

Basidiomata annual, resupinate, effused, closely adnate, thin, at first as irregular small patches, later confluent up to 7.5 cm long, 1.5 cm wide. Hymenophore surface smooth, hispidulous under lens due to the projecting cystidia (lyocystidia), white to gray, finely cracked; margin undifferentiated. Hyphal system monomitic; generative hyphae with clamp connections, colorless, tortuous, thin-walled, moderately branched, frequently septate, loosely interwoven, 2–3 μm in diam. Lyocystidia abundant, subulate, projecting beyond hymenium, multi-rooted, colorless, thick-walled, densely encrusted with minute crystals and fibrous hyphae, inner layer(s) dextrinoid, 70–90 × 8–10 μm. Basidia subclavate with a slight median constriction, colorless, thin-walled, with 4 sterigmata and a basal clamp connection, 29–32 × 8.5–9.5 μm. Basidiospores globose, distinctly apiculate, colorless, thin-walled, occasionally guttulate, inamyloid, indextrinoid, (6–)6.6–7.8(–8.8) × (5.5–)6–7(–8) μm. L = 7.2 μm, W = 6.5 μm, Q = 1–1.2 (n = 60/2; *Chen 2742* and *Wu 0102-4*). See also Oberwinkler (1965) and Kisimova-Horovitz et al. (1997a).

**Figure 3.**
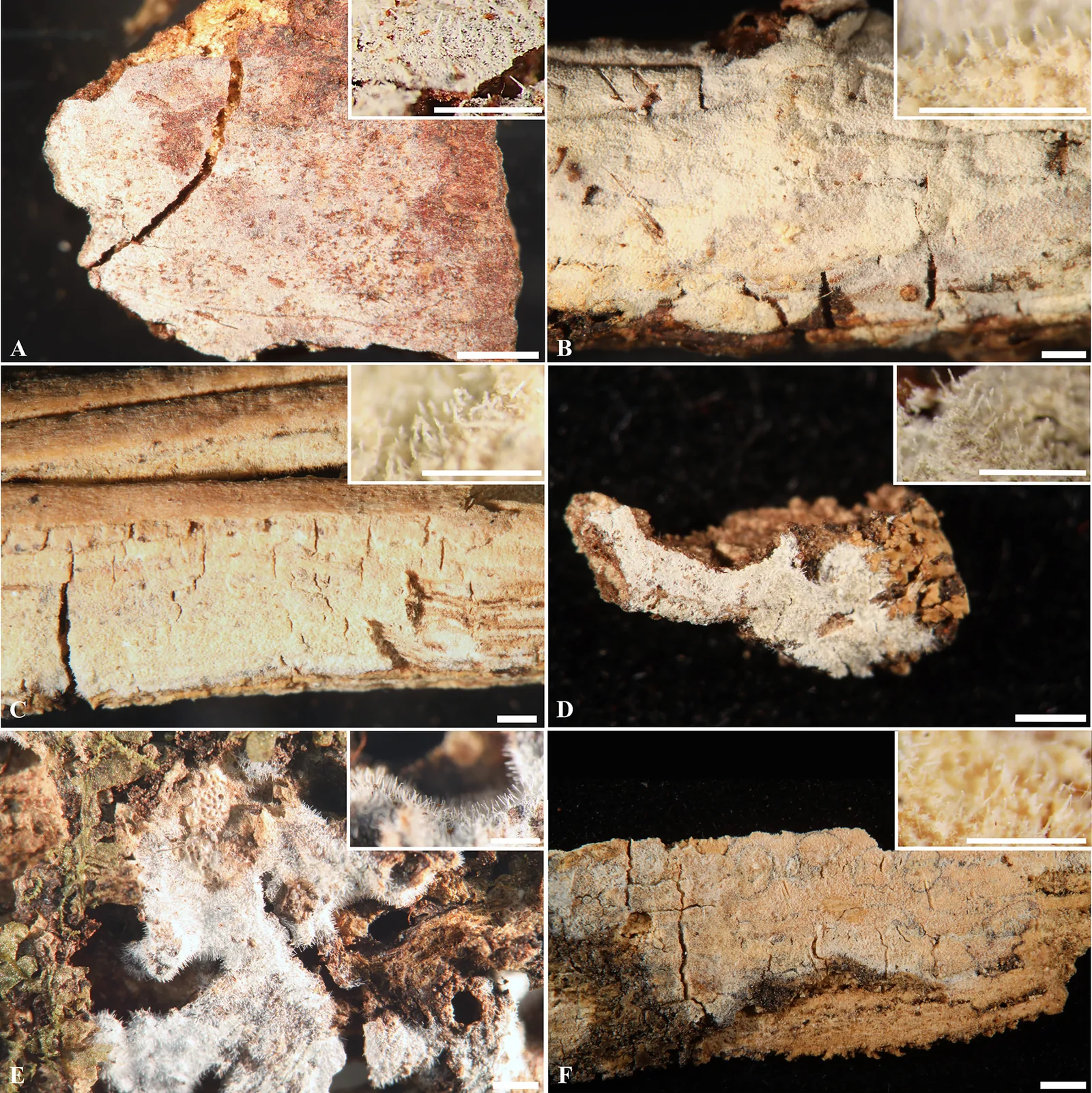
Basidiomata of *Litschauerella*. **A**. *L. abietis* (*9468*, S F259441 lectotype of *Peniophora aegerita* subsp. abietis); **B**. *L. abietis* (*Chen 2742*); **C**. *L. clematidis* (*7573*, S F16105, syntype); **D**. *L. gladiola* (PDD 11484, holotype); **E**. *L. hastata* (*GC 2406-117*); **F**. *L. hastata* (PDD 11442, holotype). Scale bars: 1 mm (bottom right) (**A–F**); 0.5 mm (upper right) (**A–F**).

**Figure 4.**
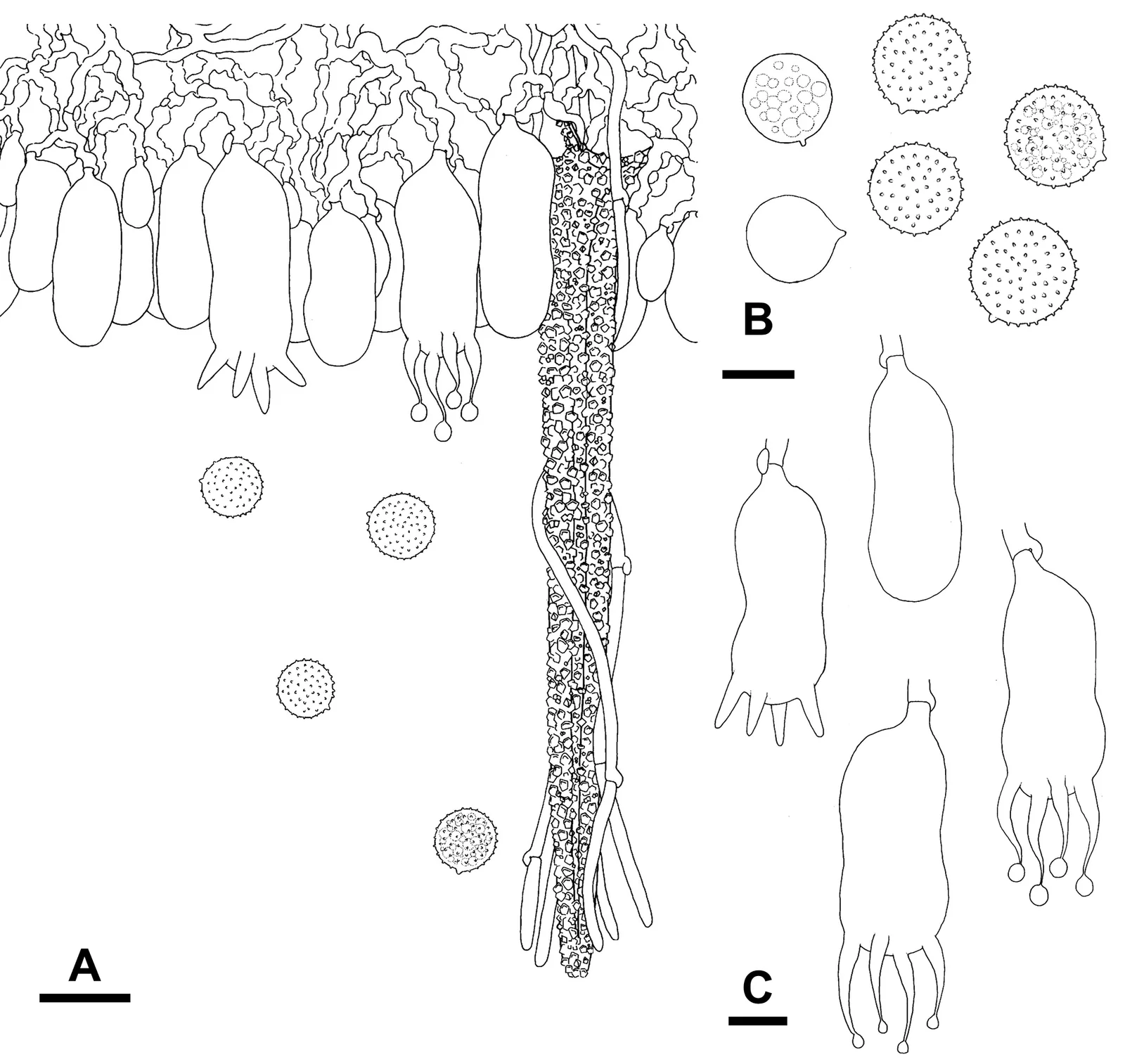
Micromorphological features of *Litschauerella abietis* (drawn from *Chen 2742*). **A**. Vertical section through basidioma; **B**. Basidiospores (two on the left in KOH; four on the right in Melzer’s reagent); **C**. Basidia and basidiole. Scale bars: 10 μm (**A**); 5 μm (**B, C**).

##### Specimens examined.

**France**. • Midi-Pyrénées, Aveyron, Arnac, on *Abies alba*, 29 May 1911, leg. A. Galzin, *9468* (S F259441 lectotype of ***P. aegerita*** subsp. **abietis**). **Taiwan**. • Nantou County, Hsinyi Township, Tatachia, 23°29'N, 120°53'E, 2450 m, on gymnosperm branch, 30 Aug 2014, leg. S.Z. Chen, W.C. Chen & C.C. Chen, *Chen 2742* (TNM F0028407). • Taipei City, Beitou District, Yangmingshan National Park, Luchiaokenghsi Ecological Reserve, 25°11'N, 121°34'E, 350 m, on angiosperm branch, 20 Feb 2001, leg. S.H. Wu & S.Z. Chen, *Wu 0102-4* (TNM F0012773). **United Kingdom**. • Devon, Slapton, Strete Gate, on old fallen *Cupressus* bark, 26 Nov 1994, leg. P. Roberts, *NA* (TNM F0003200).

##### Ecology and distribution.

Mostly on gymnosperm branches, rarely on angiosperms (Gómez 1972). Widely distributed in both hemispheres (Oberwinkler 1965; Kisimova-Horovitz et al. 1997a; Tellería et al. 2009; Chikowski et al. 2020) and Taiwan (new record; this study).

##### Notes.

The relationship between *Litschauerella abietis* and *L. clematidis* has long been debated. Earlier treatments considered *L. abietis* conspecific with *L. clematidis* due to an incorrect lectotypification of *Peniophora clematidis* Bourdot & Galzin (the basionym of *L. clematidis*) by Weresub (1961) and the nomenclatural priority of *L. clematidis* (Eriksson and Ryvarden 1976; Gilbertson and Blackwell 1987). However, Oberwinkler (1965) re-examined both taxa and separated them based on differences in basidial development (pleurobasidia versus terminal basidia) and the size of basidia and basidiospores, a view later supported by Gómez (1972). Nevertheless, the reliability of basidial development as a distinguishing feature has been questioned, as later studies (e.g., Boidin and Gilles 1989) observed that both terminal basidia and pleurobasidia can be produced within the same species. As discussed under *L. clematidis*, despite the morphological plasticity in this complex, our re-examination of type materials and additional collections supports recognizing them as distinct species. *Litschauerella abietis* generally has larger basidiospores (6–9 × 6–9 μm; Oberwinkler 1965; Kisimova-Horovitz et al. 1997a; this study) than the typical form of *L. clematidis* (4–5 × 4–4.5 μm).

#### Litschauerella clematidis

Taxon classificationFungiHymenochaetalesLitschauerellaceae

(Bourdot & Galzin) J. Erikss. & Ryvarden, Cortic. N. Eur. 4:839. 1976

919B1734-F33E-593D-8907-0F713F86250F

Fig. 3C

≡ Peniophora clematidis Bourdot & Galzin, Bull. Soc. Mycol. France 28:383. 1913.

##### Descriptions and illustrations.

See Eriksson and Ryvarden (1976) and Bernicchia and Gorjón (2010).

##### Specimens examined.

**France**. • Midi-Pyrénées, Aveyron, Nov 1910, on *Clematis*, leg. A. Galzin. *7573* (S F16105, **syntype**). **United Kingdom**. • Devon, Torquay, Watcombe Woods, on old *Clematis* vines, 5 Nov 1994, leg. P. Roberts (TNM F0003201).

##### Ecology and distribution.

Mostly on stems of *Clematis*, rarely on other angiosperm or gymnosperm hosts (Bourdot and Galzin 1912, 1928; Liberta 1960; Bernicchia et al. 2007; Bernicchia and Gorjón 2010). Distributed in both hemispheres (Bernicchia et al. 2007; Bitew and Ryvarden 2011; Chikowski et al. 2020).

##### Notes.

This species resembles *L. abietis* in general micromorphology but differs by its typically smaller basidiospores (4–5 × 4–4.5 μm) and its preference for *Clematis* hosts. *Litschauerella clematidis* is recognized as a variable species complex. As first reported by Boidin (1958) and later discussed by Weresub (1961) and Boidin and Gilles (1989), it exhibits heterosporicity, with basidiospore size varying within a single specimen from normal small basidiospores to giant spores (up to 22 μm), occasionally accompanied by the co-occurrence of large pleurobasidia and small terminal basidia; this phenomenon has been suggested to be linked to polyploidy (Boidin 1958). SEM studies by Eriksson and Ryvarden (1976) further demonstrated that basidiospore ornamentation is polymorphic, ranging from widely spaced, flat-topped warts to thin erect plates, or sometimes appearing nearly smooth.

In East Asia, Maekawa (1993) described *Litschauerella clematidis* var. *macrospora* N. Maek. from Okinawa, Japan, based on a single specimen, characterized by consistently large, distinctly verrucose, globose basidiospores (14.5–18.5 × 12–16 μm), in contrast to the occasional production of large spores in the type variety due to heterosporicity. The European specimens examined in this study conform to the traditional concept of the type variety with smaller basidiospores.

The morphological plasticity, variable spore ornamentation, and occurrence of regional variants within the *L. clematidis* complex suggest that further phylogenetic studies are needed to clarify its species boundaries.

#### Litschauerella gladiola

Taxon classificationFungiHymenochaetalesLitschauerellaceae

(G. Cunn.) Stalpers & P.K. Buchanan, New Zealand J. Bot. 29:334. 1991

F50A1ED5-D720-5C47-9B7A-139AA7594107

Fig. 3D

##### Description and illustration.

See Stalpers and Buchanan (1991).

##### Specimen examined.

**New Zealand**. • Auckland, Taneatua Reserve, 36°50'34"S, 174°45'52"E, 4 m, on *Brachyglottis repanda*, 23 May 1952, leg. G.H. Cunningham (PDD 11484, **holotype**).

##### Ecology and distribution.

Mostly on angiosperms, rarely on ferns (Spirin and Ryvarden 2016). Widely distributed in both hemispheres (Stalpers and Buchanan 1991; Ranadive et al. 2011; Spirin and Ryvarden 2016; Liu et al. 2019b).

##### Notes.

*Litschauerella gladiola* differs from other species of the genus by its basidiospores bearing inconspicuous (appearing smooth under light microscopy) or smaller, more closely packed (under SEM) warts (Stalpers and Buchanan 1991).

#### Litschauerella hastata

Taxon classificationFungiHymenochaetalesLitschauerellaceae

(G. Cunn.) Stalpers & P.K. Buchanan, New Zealand J. Bot. 29:336. 1991

E5E48341-6050-58AC-8C39-7AF835762A05

Fig. 3E, F

##### Description and illustration.

See Stalpers and Buchanan (1991).

##### Specimens examined.

**New Zealand**. • Auckland, Piha, Glen Esk Valley, on *Griselinia lucida* Forst. f., 12 May 1951, leg. J.M. Dingley (PDD 11442, **holotype**). **Vietnam**. • Lâm Đồng Province, Hon Giao Forestry Ranger Station, mossy forest, 12°11'07"N, 108°43'01"E, 1700 m, on rotting trunk, 19 Jun 2024, leg. C.C. Chen, *GC 2406-117* (TNM F0039302).

##### Ecology and distribution.

On angiosperms in New Zealand (Cunningham 1955; Stalpers and Buchanan 1991) and Vietnam (new record; this study).

##### Notes.

*Litschauerella hastata* is distinguished from other species of the genus by its subglobose to broadly ellipsoid basidiospores (6–8.5 × 5–6 μm; measured from listed specimens).

### *Trechisporales* K.H. Larss., in Hibbett et al., Mycol. Res. 111:541. 2007

***Hydnodontaceae* Jülich, Bibl. Mycol. 85:372. 1982**

#### Tubulicium

Taxon classificationFungiTrechisporalesHydnodontaceae

Oberw., Sydowia 19:53. 1966

6F184BC5-A993-50FA-93F5-157A48D2D555

##### Notes.

*Tubulicium* is phylogenetically sister to the monotypic genus *Membranohymenium* within the *Trechisporales* (Fig. 2). Both genera share resupinate basidiomata and possess cystidia that have been interpreted as lyocystidia or lyocystidia-like structures. However, *Tubulicium* differs from *Membranohymenium* in having a smooth (vs. grandinioid) hymenial surface, and fusiform to vermicular basidiospores (vs. cylindrical to allantoid, 6.5–10.5 × 1.5–3 μm in *M. grandinioides*; Wijesinghe et al. 2025).

#### Tubulicium bambusicola

Taxon classificationFungiTrechisporalesHydnodontaceae

S.H. He & S.L. Liu, MycoKeys 48:108. 2019

CB612AB3-894D-5909-A697-6B62B6A661C6

Fig. 5A

##### Description and illustration.

See Liu et al. (2019b).

**Figure 5.**
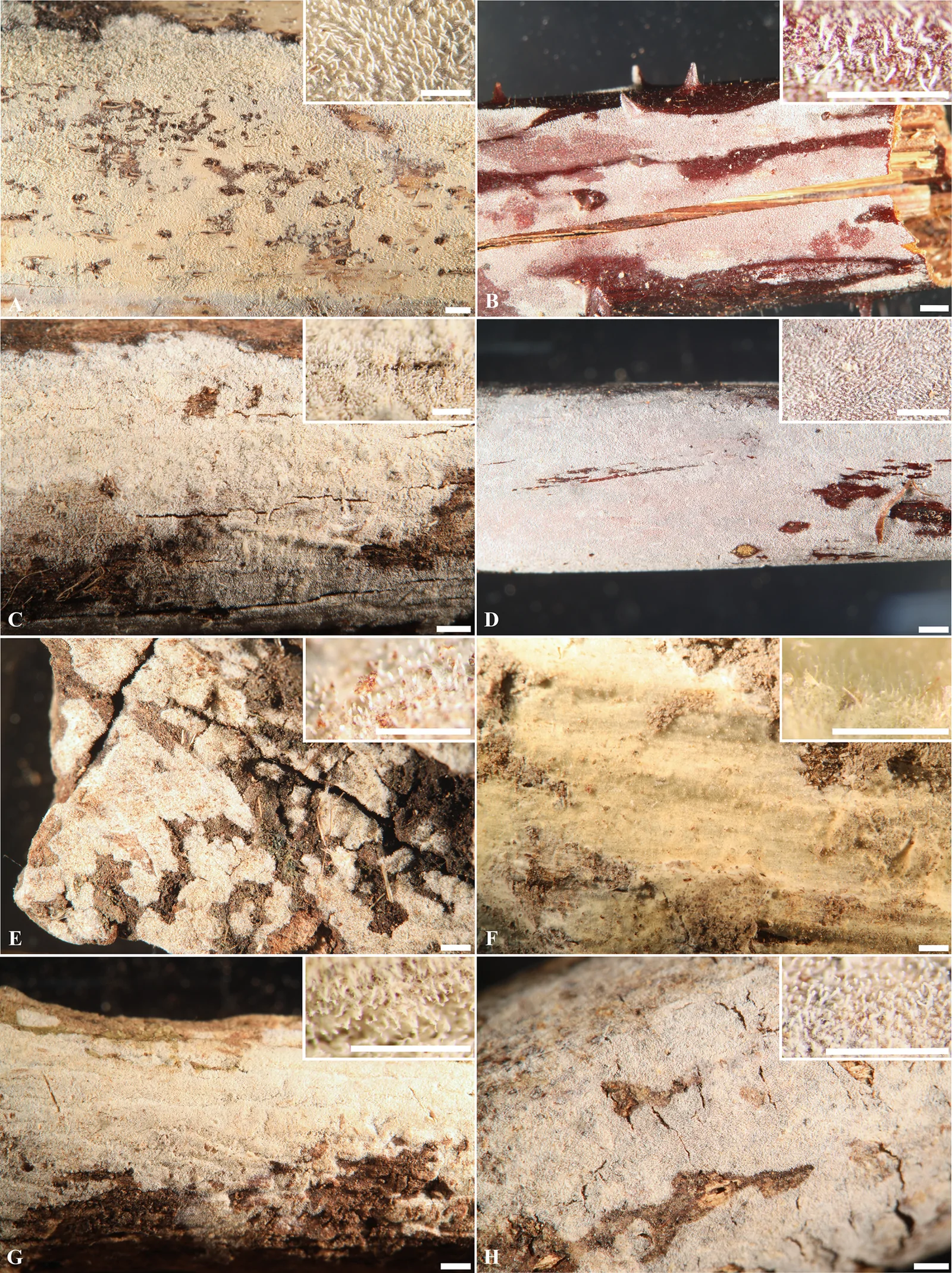
Basidiomata of *Tubulicium*. **A**. *T. bambusicola* (*Chen 1731*); **B**. *T. curvisporum* (*WEI 19-095*); **C**. *T. densum* (*WEI 17-045*, holotype); **D**. *T. dussii* (*WEI 19-508*); **E**. *T. fusiforme* (*Wu 0107-2*, holotype); **F**. *T. naviculisporum* (*WEI 18-615*, holotype); **G**. *T. raphidisporum* (*Wu 9806-9*); **H**. *T. vermiferum* (*Wu 901125-15*). Scale bars: 1 mm (bottom right) (**A–H**); 0.5 mm (upper right) (**A–H**).

##### Specimens examined.

**China**. • Yunnan Province, Xishuangbanna, Menglun Tropical Botanical Garden, 21°56'N, 101°15'E, 500 m, on bamboo culm, 11 Aug 1995, leg. S.H. Wu & J.Y. Tseng, *Wu 9508-204* (TNM F0004115); • ibid., Menghai, Nannoshan, 21°57'N, 100°35'E, 1400 m, on bamboo culm, 15 Aug 1995, leg. S.H. Wu & J.Y. Tseng, *Wu 9508-270* (TNM F0004176); • ibid., Napanho, 22°8'N, 100°40'E, 600 m, 16 Aug 1995, *Wu 9508-287* (TNM F0004193); • ibid., *Wu 9508-303* (TNM F0004209). **Taiwan**. • Kaohsiung City, Sanming Township, 23°11'N, 120°39'E, 580 m, on bamboo culm, 23 Dec 1993, leg. S.H. Wu & S.Z. Chen, *Wu 9312-85* (TNM F0001584). • Pingtung County, the vicinity of Kenting, 21°57'N, 120°48'E, 20 m, on dead bamboo stem, 21 Aug 2011, leg. E.O. Yurchenko, *EYu 110821-1* (TNM F0024971). • Taichung City, Hoping District, 5.5 km of Tungmaoshan Trail, 24°11'21"N, 120°57'26"E, 1200 m, on *Arenga engleri* stem, 29 Oct 2019, leg. C.L. Wei, *WEI 19-403* (TNM F0036244). • Taitung County, Green Island, Youzihhu, 22°40'N, 121°30'E, 35 m, on bamboo culm, 9 Jun 2010, leg. S.Z. Chen, *Chen 1720* (TNM F0025034); • ibid., *Chen 1721* (TNM F0025035); • ibid., *Chen 1731* (TNM F0025045); • ibid., Tunghe Township, Dulan Mountain Trail, 22°52'N, 121°11'E, 650 m, on bamboo, 21 Jul 2017, leg. S.Z. Chen & Y.Y. Huang, *Chen 3627* (TNM F0032396).

##### Ecology and distribution.

On bamboo culms and occasionally on stems of *Arenga engleri*; China, Thailand (Liu et al. 2019b), and Taiwan (new record; this study). Jun to Aug, Dec.

##### Note.

*Tubulicium bambusicola* is distinguished by its narrowly fusiform to vermicular basidiospores and frequent occurrence on bamboo. One Taiwanese specimen (*WEI 19-403*) occurred on *Arenga engleri*, suggesting the host range may be broader. *T. raphidisporum* and *T. vermiferum* are similar in having long, fusiform to vermiform basidiospores but differ by their wider basidiospores (≥ 3.5 μm) and preference for woody substrates (Maekawa 1998; Bernicchia and Gorjón 2010). It may also be confused with *T. densum*, *T. fusiforme* and *T. naviculisporum*; see notes under each taxon.

#### Tubulicium curvisporum

Taxon classificationFungiTrechisporalesHydnodontaceae

Ushijima & N. Maek., Mycoscience 60:138, 2019

9DB18429-690F-53EE-9F79-51E9CB7D8AE8

Fig. 5B

##### Description and illustration.

See Ushijima et al. (2019).

##### Specimens examined.

**Japan**. • Kagoshima Prefecture, Kumage District, Yaku Island, 200 m, on a decaying petiole of *Cyathea fauriei*, 27 Oct 1995, leg. N. Maekawa, *19744* (TNM F0005029). **Taiwan**. • New Taipei City, Wulai District, Laka Hiking Trail, 24°51'N, 121°33'E, 360 m, on tree fern, 24 Jan 2019, leg. C.C. Chen & C.L. Wei, *WEI 19-095* (TNM F0034809).

##### Ecology and distribution.

On living *Pieris japonica* or tree ferns in Japan (Ushijima et al. 2019) and Taiwan (new record; this study). Mar, Jun, Jul, Sep to Nov.

##### Note.

Ecologically, *T. curvisporum* frequently grows on Japanese andromeda (*Pieris japonica*) (Ushijima et al. 2019). However, we found two specimens (*19744* and *WEI 19-095*) on tree fern in Japan and Taiwan, respectively. These observations indicate that the species may have a wider host range than previously documented.

Morphologically, *Tubulicium curvisporum* resembles *T. dussii* and *T. junci-acuti* in having a smooth hymenophore, distinctive lyocystidia, curved basidiospores, and in growing on ferns. However, *T. dussii* differs by its smaller basidiospores (*T. dussii*: 6.5–7.5 × 2.5–3 μm; *T. curvisporum*: 16.5–25.5 × 4.5–7 μm) (Dämon and Hausknecht 2003; Ushijima et al. 2019), whereas *T. junci-acuti* has narrower basidiospores that are often slightly constricted halfway (*T. junci-acuti*: 15–20 × 3–4.3 μm; Boidin and Gaignon 1992).

#### Tubulicium densum

Taxon classificationFungiTrechisporalesHydnodontaceae

Yi C. Lin & C. Chih Chen sp. nov.

49BC05F6-CF00-5A17-86B9-80054861F381

860277

Figs 5C, 6

##### Etymology.

densum, refers to the dense crystal encrustations that cover the lyocystidia.

**Figure 6.**
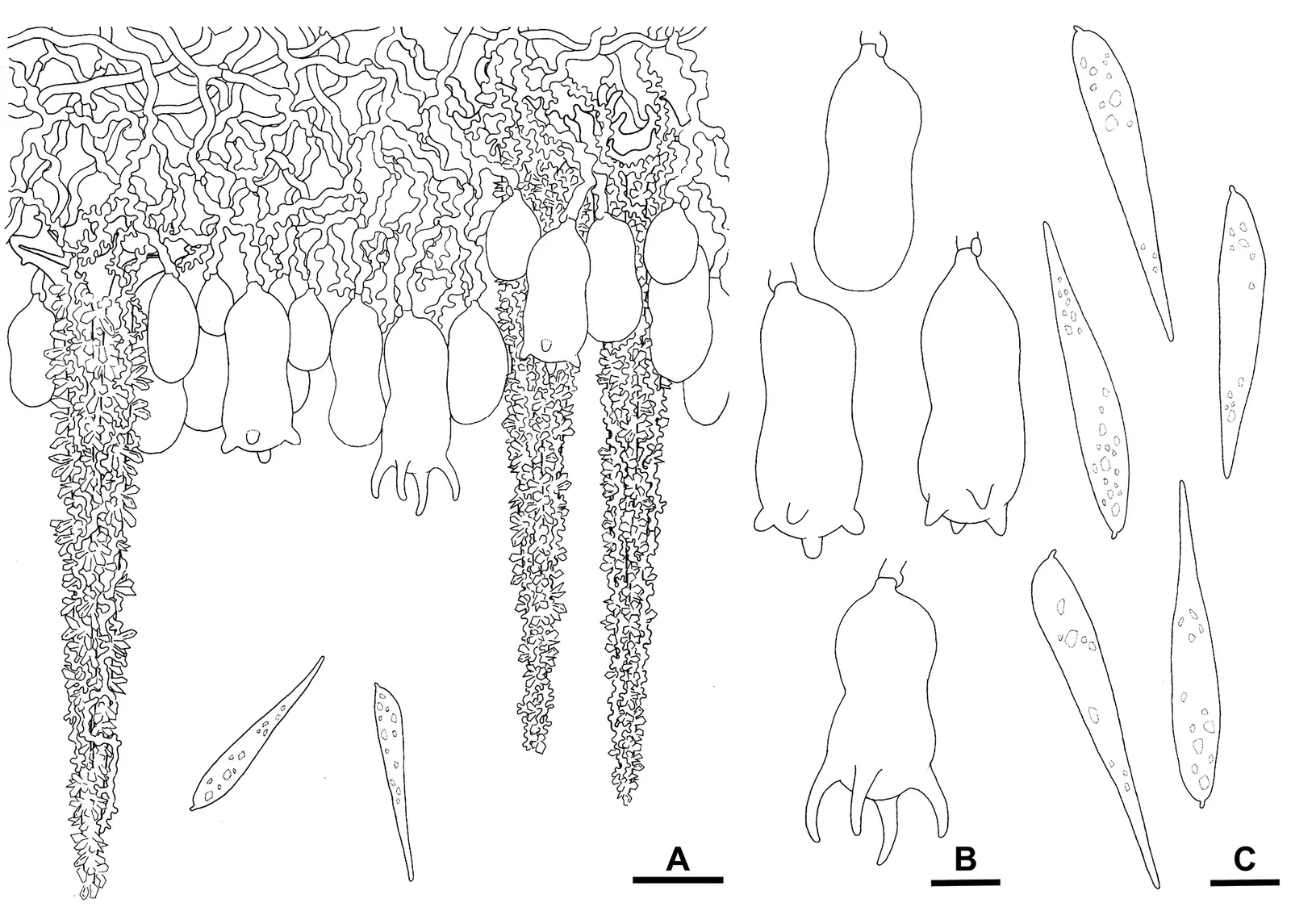
Micromorphological features of *Tubulicium densum* (drawn from *WEI 17-045*, holotype). **A**. Vertical section through basidioma; **B**. Basidia and basidiole; **C**. Basidiospores. Scale bars: 10 μm (**A**); 5 μm (**B, C**).

##### Diagnosis.

*Tubulicium densum* is characterized by white to cream basidiomata with a smooth hymenophore, lyocystidia bearing dendroidly branching hyphae and columnar crystals, and oily, fusiform basidiospores.

##### Typification.

**Taiwan**. • Nantou County, Jenai Township, Huisun Forestry Station, 24°5'N, 121°2'E, 750 m, on angiosperm branch, 24 Mar 2017, leg. S.H. Wu, C.C. Chen, C.L. Wei, & Y.T. Lin, *WEI 17-045* (**holotype**TNM F0031826). GenBank: ITS = PX060166; 28S = PX048511.

##### Description.

Basidiomata annual, resupinate, adnate, thin, at first as irregular small patches, later confluent up to 10 cm long, 2 cm wide. Hymenophore surface smooth, hispidulous under lens due to the projecting cystidia (lyocystidia), white to cream, finely cracked; margin undifferentiated. Hyphal system monomitic; generative hyphae with clamp connections, colorless, tortuous, thin-walled, moderately branched, frequently septate, loosely interwoven, 1.5–2.5 μm in diam. Lyocystidia abundant, subulate, projecting beyond hymenium, multi-rooted, colorless, distinctly thick-walled, covered with dendroidly branching hyphae and columnar crystals, 65–90 × 7–10 μm, negative to weakly amyloid in Melzer’s reagent. Basidia subclavate with a slight median constriction, colorless, thin-walled, with 4 sterigmata and a basal clamp connection, 21–27 × 6–8 μm. Basidiospores narrowly fusiform to navicular, biapiculate, colorless, thin-walled, smooth, oily, inamyloid, indextrinoid, (19–)20.5–26(–28.5) × (2.8–)3–3.5(–4.1) μm. L = 23.3 μm, W = 3.3 μm, Q = 6.4–7.9 (n = 30).

##### Additional specimens examined.

**China**. • Yunnan Province, Xishuangbanna, Menglun Nature Reserve, road No. 53, 21°58'N, 101°12'E, 600 m, on angiosperm branch, 12 Aug 1995, leg. S.H. Wu & J.Y. Tseng, *Wu 9508-226* (TNM F0004134). **Taiwan**. • Taichung City, Hoping Township, Henglingshan Trail, 24°14'N, 120°56'E, 1770 m, on angiosperm branch, 7 May 2017, leg. C.L. Wei, *WEI 17-123* (TNM F0031870); • ibid., Kukuan, 24°13'N, 121°1'E, 760 m, on dead fallen liana stem, 15 May 2011, leg. E.O. Yurchenko, *EYu 110515-19* (TNM F0024910); • ibid., on fallen branch among stone conglomeration, *EYu 110515-6* (TNM F0024902). • Taitung County, Orchid Island, between trash treatment field and Tienchi, 22°1'N, 121°34'E, 150 m, on angiosperm branch, 1 May 1997, leg. S.H. Wu & J.Y. Tseng, *Wu 9705-19* (TNM F0008742); • ibid., *Wu 9705-28* (TNM F0008751); • ibid., *Wu 9705-38* (TNM F0008761). • Taipei City, Beitou District, Mifengchao Historical Trail, 25°11'N, 121°32'E, 740 m, on angiosperm branch, 3 Dec 2019, leg. C.L. Wei, *WEI 19-509* (TNM F0036440).

##### Ecology and distribution.

On angiosperm branches in SW China (Yunnan) and Taiwan. Mar, May to Aug, Dec.

##### Note.

Phylogenetically (Fig. 2), the ITS sequence of the holotype (*WEI 17-045*) is 99% similar (549/555 bp) to OTU73 (GenBank: MH005912), an environmental sequence detected from the mycorrhizal roots of an epiphytic orchid in China (Xing et al. 2019). This suggests a possible ecological association that merits further investigation. In addition, *T. densum* forms a clade closely related to *T. bambusicola* and *T. fusiforme*.

Morphologically, *T. densum* can be distinguished from *T. bambusicola* by the presence of crystal encrustations on its lyocystidia (Liu et al. 2019b). It differs from *T. fusiforme* by having columnar (rather than polytetrahedral) crystals and narrower basidiospores (3–3.5 µm wide). While *T. densum* resembles *T. raphidisporum* and *T. vermiferum* in having smooth hymenophore and fusiform to vermiform basidiospores, it differs from *T. raphidisporum* by its smaller, encrusted cystidia (vs. 90–160 × 11–16 µm, without crystals) and shorter basidiospores (vs. 27–35 × 3–4 µm; Maekawa 1998). Additionally, *T. vermiferum* can be separated from *T. densum* by its broader basidiospores (vs. 20–25 × 3.5–4 µm; Bernicchia and Gorjón 2010).

#### Tubulicium dussii

Taxon classificationFungiTrechisporalesHydnodontaceae

(Pat.) Oberw. ex Jülich, Persoonia 10:335. 1979

6410CC8D-0C7D-55F1-9006-B9AD3868D662

Fig. 5D

= Tubulicium vermiculare (Wakef.) Boidin & Gilles, Bull. trimest. Soc. mycol. Fr. 102:282. 1986.

##### Description and illustration.

See Dämon and Hausknecht (2003).

##### Specimens examined.

**Taiwan**. • Taipei City, Yangmingshan National Park, Luchiaokenghsi Ecological Reserve, 25°11'N, 121°34'E, 350 m, on culm of *Daemonorops margaritae*, 20 Feb 2001, leg. S.H. Wu & S.Z. Chen, *Wu 0102-6* (TNM F0012775); • ibid., Beitou District, Mifengchao Historical Trail, 25°11'N, 121°32'E, 740 m, on tree fern, 3 Dec 2019, leg. C.L. Wei, *WEI 19-508* (TNM F0034961). • Nantou County, Hsitou, 1150 m, on tree fern, 29 Jun 2008, leg. S.H. Wu, *Wu 0806-2* (TNM F0022044).

##### Ecology and distribution.

On tree ferns (e.g., *Dicksonia antarctica*, *Daemonorops margaritae*) in subtropical regions of both hemispheres, including Portugal, Guadaloupe Island, Madeira Island, Costa Rica, New Zealand (Jülich 1979; Kisimova-Horovitz et al. 1997b; Dämon and Hausknecht 2003; Telleria et al. 2008), and Taiwan (new record; this study). Feb, Jun, Dec in Taiwan.

##### Notes.

*Tubulicium dussii* is distinguished by smooth hymenophore, distinctive lyocystidia, sublunate to navicular basidiospores, and in growing on ferns (Dämon and Hausknecht 2003). It is similar to *T. curvisporum*; a detailed comparison is provided in the notes of *T. curvisporum*.

#### Tubulicium fusiforme

Taxon classificationFungiTrechisporalesHydnodontaceae

Yi C. Lin & C. Chih Chen sp. nov.

504588BC-540C-56A4-BFCD-F3FFBF9199C5

860279

Figs 5E, 7

##### Etymology.

fusiforme, refers to the fusiform shape of the basidiospores.

**Figure 7.**
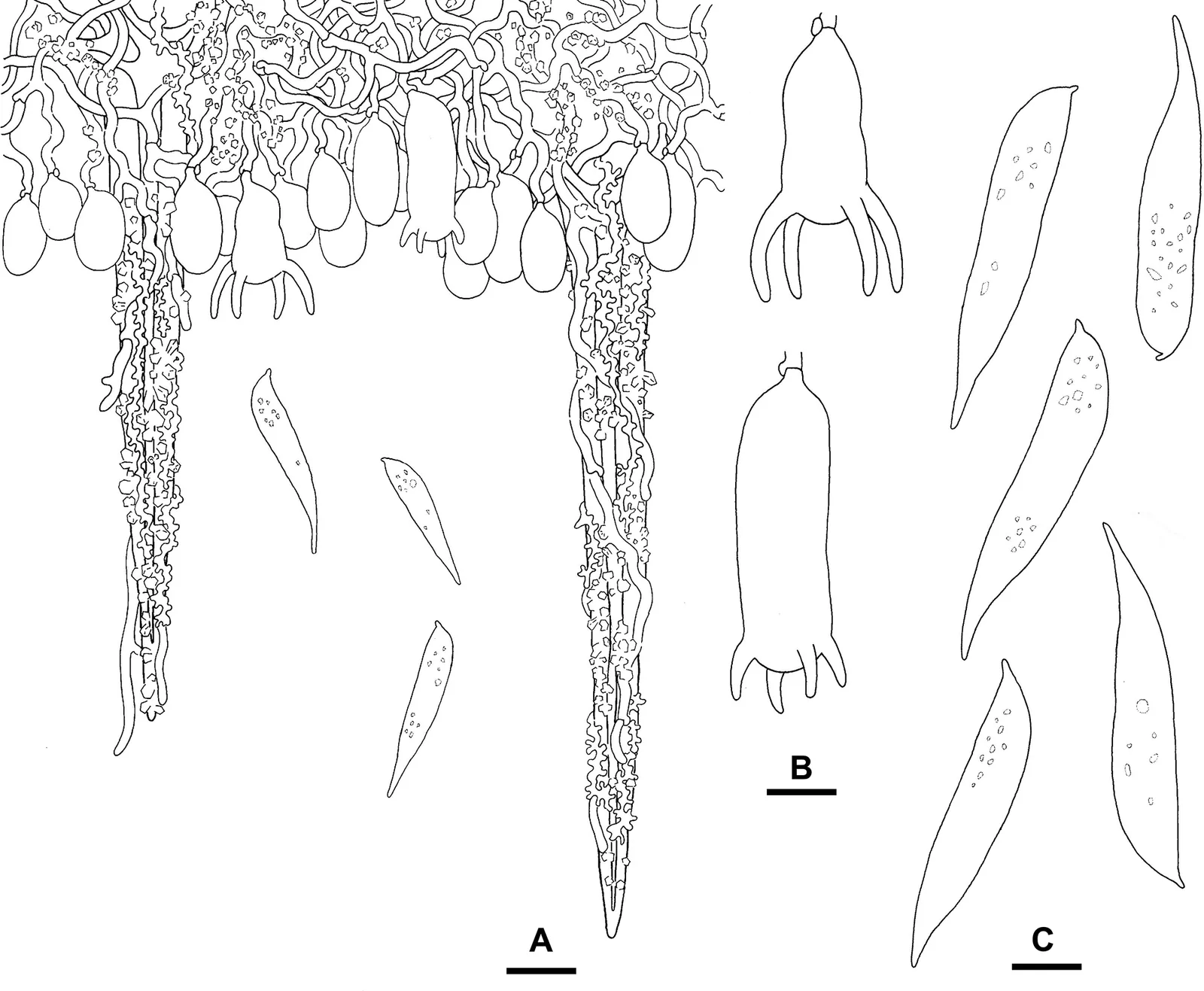
Micromorphological features of *Tubulicium fusiforme* (drawn from *Wu 0107-2*, holotype). **A**. Vertical section through basidioma; **B**. Basidia; **C**. Basidiospores. Scale bars: 10 μm (**A**); 5 μm (**B, C**).

##### Diagnosis.

*Tubulicium fusiforme* is distinguished by white to cream basidiomata with a smooth hymenophore, lyocystidia bearing dendroidly branching or cylindrical hyphae and polytetrahedral crystals, and oily, fusiform and slightly vermicular basidiospores.

##### Typification.

**China**. • Yunnan Province, Heilungtan, Botanical Garden. 25°8'N, 102°44'E, 1850 m, on rotten trunk of *Eucalyptus maideni*, 24 Jul 2001, leg. S.H. Wu & S.Z. Chen, *Wu 0107-2* (holotype TNM F0013489). GenBank: ITS = PX060164.

##### Description.

Basidiomata annual, resupinate, adnate, thin, forming irregular small patches. Hymenophore surface smooth, hispidulous under lens due to the projecting cystidia (lyocystidia), white to cream, finely cracked; margin undifferentiated. Hyphal system monomitic; generative hyphae with clamp connections, colorless, thin-walled, moderately branched, moderately septate, loosely interwoven, 1.5–2.5 μm in diam. Lyocystidia abundant, subulate, projecting beyond hymenium, multi-rooted, colorless, distinctly thick-walled, covered with dendroidly branching hyphae, cylindrical hyphae and polytetrahedral crystals, 90–110 × 10–14 μm, negative to weakly amyloid. Basidia subclavate with a slight median constriction, colorless, thin-walled, with 4 sterigmata and a basal clamp connection, 21–27 × 8–9.5 μm. Basidiospores fusiform to navicular, slightly vermicular, biapiculate, colorless, thin-walled, smooth, oily, inamyloid, indextrinoid, (21.5–)23–26(–27.2) × (3.9–)4–4.4(–4.5) μm. L = 24.3 μm, W = 4.2 μm, Q = 4.9–6.7 (n = 30).

##### Additional specimens examined.

**Italy**. • Latina Province, Circeo National Park, Selva di Circe, on *Eucalyptus*, 22–25 Oct 1984, leg. K. Hjortstam et al., *Hjm 14872* (TNM F0016809). **Taiwan**. • Chiayi County, Alishan Township, Yushan Forestry Trail, 23°28'20"N, 120°50'50"E, 2350 m, on angiosperm branch, 3 Jun 2019, leg. C.C. Chen & C.L. Wei, *WEI 19-243* (TNM F0035559). • Taipei City, Wulai District, Neidong National Forest Recreation Area, 24°50'N, 121°32'E, 280 m, on dead bases of petioles of living palm, 23 Jun 2011, leg. E.O. Yurchenko, *EYu 110623-14* (TNM F0024863).

##### Ecology and distribution.

On branches of *Eucalyptus* or other angiosperms, also on dead petioles of living palms; SW China (Yunnan), Italy, and Taiwan. Jun, Jul, and Oct.

##### Note.

Phylogenetically (Fig. 2), the ITS sequence of the holotype (*Wu 0107-2*) is 99% similar (307/310 bp) to MYL7403 (GenBank: LC697296), an environmental sequence detected from healthy plant materials of tea (*Camellia sinensis*) in Japan (Rungjindamai and Jones, unpublished). In our analysis, the ITS sequence of *T. fusiforme* is 96% similar to *T. bambusicola* (518/540 bp) and 96% similar to *T. densum* (507/526 bp).

Morphologically, *T. fusiforme* is closely related to *T. bambusicola* and *T. densum*, but differs from *T. bambusicola* by the presence of crystals on the cystidia (Liu et al. 2019b), and from *T. densum* by having polytetrahedral crystals on cystidia and wider basidiospores (vs. 3–3.5 μm in *T. densum*). It may be confused with *T. raphidisporum*, and *T. vermiferum*, but differs by having wider basidiospores than both, and the presence of polytetrahedral crystals on cystidia (Fig. 7A; Maekawa 1998; Bernicchia and Gorjón 2010).

#### Tubulicium naviculisporum

Taxon classificationFungiTrechisporalesHydnodontaceae

Yi C. Lin & C. Chih Chen sp. nov.

30D5D03F-3B5E-5526-A1E7-554BA450962D

860278

Figs 5F, 8

##### Etymology.

naviculisporum, refers to the navicular (boat-shaped) basidiospores of this species.

**Figure 8.**
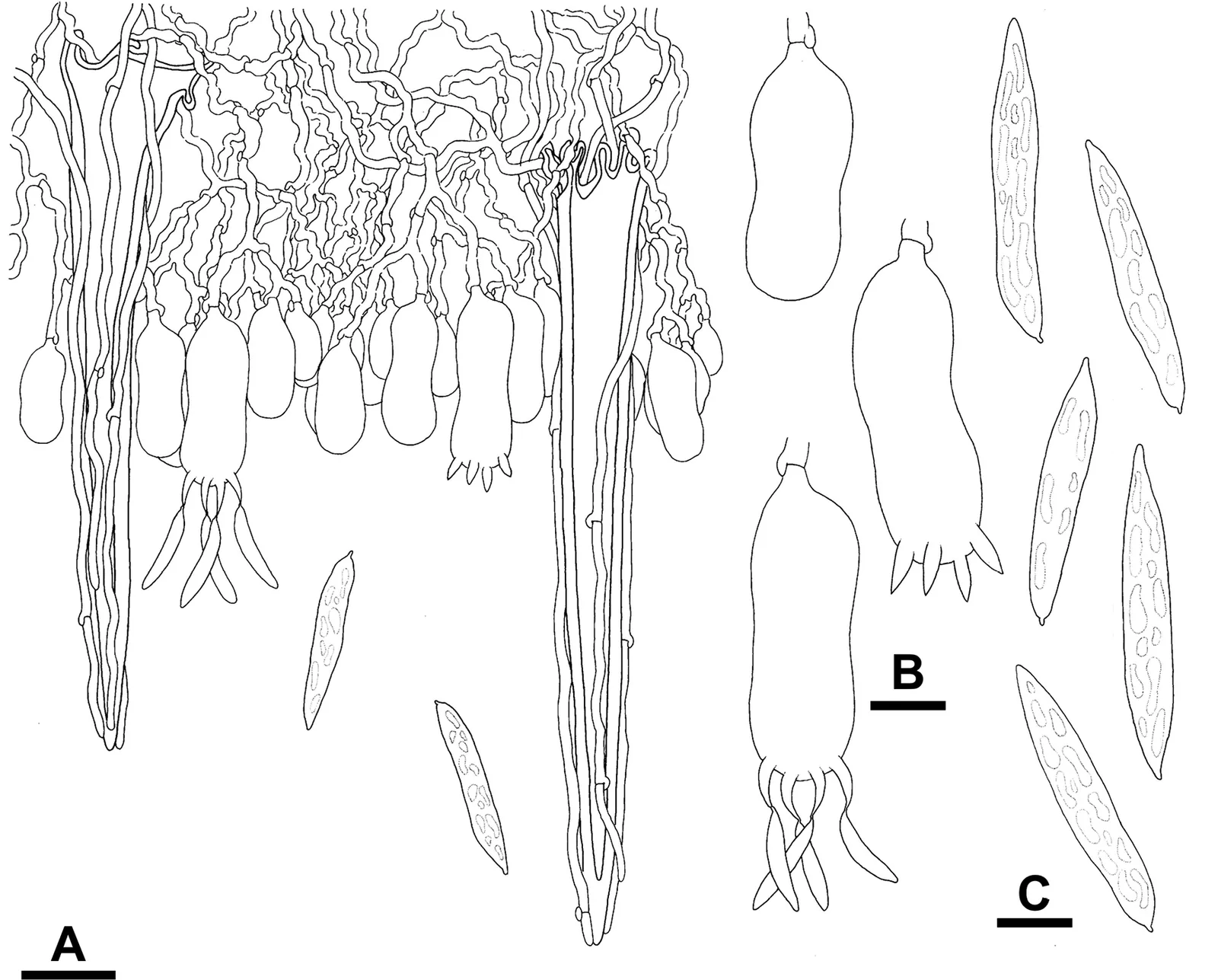
Micromorphological features of *Tubulicium naviculisporum* (drawn from *WEI 18-615*, holotype). **A**. Vertical section through basidioma; **B**. Basidia and basidiole; **C**. Basidiospores. Scale bars: 10 μm (**A**); 5 μm (**B, C**).

##### Diagnosis.

*Tubulicium naviculisporum* is characterized by white to cream basidiomata with smooth hymenophore, lyocystidia covered with cylindrical hyphae, and guttulate, navicular to fusiform basidiospores.

##### Typification.

**Taiwan**. • Pingtung County, Manchou Township, Chufengshan, 22°2'N, 120°51'E, 90 m, on angiosperm branch, 29 Nov 2018, leg. C.C. Chen & C.L. Wei, *WEI 18-615* (holotype TNM F0034709). GenBank: ITS = PX060167; 28S = PX048509.

##### Description.

Basidiomata annual, resupinate, adnate, thin, at first as irregular small patches, later confluent up to 11 cm long, 2 cm wide. Hymenophore surface smooth, hispidulous under lens due to the projecting cystidia (lyocystidia), white to cream, finely cracked; margin undifferentiated. Hyphal system monomitic; generative hyphae with clamp connections, colorless, more or less tortuous, indistinct, thin-walled, moderately branched, frequently septate, loosely interwoven, 1.5–2.5 μm in diam. Lyocystidia abundant, subulate, projecting beyond hymenium, multi-rooted, colorless, distinctly thick-walled, covered with cylindrical hyphae, slightly amyloid, 70–90 × 6–11 μm. Basidia subclavate with a slight median constriction, colorless, thin-walled, with 4 sterigmata and a basal clamp connection, 22–28 × 6–8 μm. Basidiospores navicular to fusiform, biapiculate, colorless, thin-walled, smooth, guttulate, inamyloid, indextrinoid, (17–)18.5–22.5(–24.5) × (3–)3.2–3.8(–4.1) μm. L = 20.7 μm, W = 3.5 μm, Q = 5.3–6.5 (n = 30).

##### Additional specimen examined.

**Taiwan**. • Pingtung County, Manchou Township, Nanjenshan, 22°5'N, 120°52'E, 320 m, on angiosperm branch, 28 Nov 2018, leg. C.C. Chen & C.L. Wei, *WEI 18-580* (TNM F0034494).

##### Ecology and distribution.

On angiosperm branches in Taiwan. Nov.

##### Note.

*Tubulicium naviculisporum* resembles *T. bambusicola*, *T. raphidisporum*, and *T. vermiferum* in having a smooth hymenophore and fusiform to vermiform basidiospores. However, it differs by having cystidia covered with cylindrical rather than dendroidly branching hyphae and by its navicular basidiospores that do not taper to a single acute end (Kisimova-Horovitz et al. 1997b; Hjortstam et al. 1998; Liu et al. 2019b).

#### Tubulicium raphidisporum

Taxon classificationFungiTrechisporalesHydnodontaceae

(Boidin & Gilles) Oberw., Kisim.-Hor. & L.D. Gómez, Revista de Biologia Tropical 45:1313. 1998

6ED57DAF-99FA-536A-ADE7-1C5FF487CC72

Fig. 5G

##### Description and illustration.

See Maekawa (1998).

##### Specimens examined.

**China**. • Yunnan Province, Yuxi City, Ailao Mountain, 24°32'24"N, 101°1'30"E, 2485 m, on angiosperm branch, 25 Aug 2015, leg. S.H. Wu, *Wu 1508-90* (TNM F0030109). **Puerto Rico**. • Caribbean National Park, 1000 m, on angiosperm branch, 11 Jun 1998, leg. S.H. Wu, *Wu 9806-9* (TNM F0010197). **Taiwan**. • Taichung City, Heping District, 43 km of Dasyueshan Forestry Road, Anma Villa, 24°15'22"N, 120°59'59"E, 2250 m, on angiosperm branch, 20 Aug 2008, leg. S.Z. Chen, Y.T. Wang & H.X. Xiong, *Wu 0808-145* (TNM F0022767); • ibid., Anmashan, 24°16'N, 121°0'E, 2250 m, on angiosperm branch, 7 Jun 1995, leg. S.H. Wu & J.Y. Tseng, *Wu 9506-47* (TNM F0003636).

##### Ecology and distribution.

On angiosperm branches in Puerto Rico, Africa, SW China (Yunnan), Japan, South America and Taiwan (Kisimova-Horovitz et al. 1997a; Maekawa et al. 2003; Hjortstam and Ryvarden 2008; Martini 2016; Liu et al. 2019b; Liu et al. 2022; this study). Jun, Aug in SW China and Taiwan.

##### Notes.

*Tubulicium raphidisporum* is distinguished by smooth hymenophore, distinctive lyocystidia with dendroidly branching hyphae, fusiform basidiospores (Kisimova-Horovitz et al. 1997a). It is similar to *T. bambusicola*; see notes of *T. bambusicola* for comparison.

#### Tubulicium vermiferum

Taxon classificationFungiTrechisporalesHydnodontaceae

(Bourdot) Oberw. ex Jülich, Persoonia 10:335. 1979

90C5F392-A6B6-5D84-B1B0-72F93C8A013F

Fig. 5H

##### Descriptions and illustrations.

See Bernicchia and Gorjón (2010) and Hjortstam et al. (1998).

##### Specimens examined.

**France**. • Pyrénées-Orientales Department, near Conat, 400 m, on *Erica arborea*, 31 Oct 1995, leg. J. Boidin, *LY 16452* (TNM F0004496). **Taiwan**. • Taipei City, Gongliao District, 25°0'N, 121°54'E, 200 m, on angiosperm branch, 25 Nov 1990, leg. S.H. Wu, *Wu 901125-15* (TNM F0015022).

##### Ecology and distribution.

On angiosperm branches (e.g., *Erica arborea*). Widely distributed in both hemispheres (Liberta 1960; Maekawa 1992; Hjortstam et al. 1998; Hjortstam and Ryvarden 2008; Bernicchia and Gorjón 2010; Kunttu et al. 2011) and Taiwan (new record; this study).

##### Notes.

*Tubulicium vermiferum* is distinguished by smooth hymenophore, distinctive lyocystidia with dendroidly branching hyphae and a crystal layer, and vermiform basidiospores (Bernicchia and Gorjón 2010). A detailed comparison has been provided above.

### Key to genera of *Litschauerella* and *Tubulicium*

**Table:** 

1	Cystidia encrusted with crystals and fibrous hyphae, inner layer weakly to strongly dextrinoid; basidiospores globose to broadly ellipsoid, ornamented in Melzer’s reagent	*** Litschauerella ***
–	Cystidia mostly encrusted with dendroidly branching hyphae, few with crystals, inner layer weakly amyloid, basidiospores ellipsoid, navicular, fusiform to vermicular, smooth in Melzer’s reagent	*** Tubulicium ***

### Global key to the species of *Litschauerella*

**Table:** 

1	Basidiospores subglobose to broadly ellipsoid, 6–8.5 × 5–6 μm	***L. hastata***
–	Basidiospores globose to subglobose	**2**
2	Basidiospores smooth to slightly ornamented in Melzer’s reagent	***L. gladiola***
–	Basidiospores distinctly ornamented in Melzer’s reagent	**3**
3	Basidiospores longer than 6 μm, 6–9 × 6–9 μm	***L. abietis***
–	Basidiospores shorter than 6 μm, 4–5 × 4–4.5 μm	***L. clematidis***

### Global key to the species of *Tubulicium*

**Table:** 

1	Basidiospores not globose (ovate to ellipsoid)	**4**
–	Basidiospores globose to subglobose	**2**
2	Basidiospores globose to subglobose (Q < 2), 15–18 × 11–12 µm	***T. erectum***
–	Basidiospores ovate to ellipsoid	**3**
3	Basidiospores ovate, apiculate, cystidia weakly amyloid	***T. ramonense***
–	Basidiospores amygdaliform, biapiculate, cystidia strongly dextrinoid	***T. papillatosporum***
4	Basidiospores up to 10 μm in length	**5**
–	Basidiospores > 10 μm in length	**6**
5	Basidiospores obtuse, cylindrical to suballantoid, 6–8 × 2 µm	***T. filicicola***
–	Basidiospores sublunate to navicular, 6.5–7.5 × 2.5–3 µm	***T. dussii***
6	Basidiospores curved (sickle-shaped, lunate, or sigmoidal)	**7**
–	Basidiospores not curved, navicular	**9**
7	Basidiospores sickle-shaped, 16.5–25.5 × 4.5–7 µm, Q < 4	***T. curvisporum***
–	Basidiospores lunate or sigmoidal, Q > 4	**8**
8	Basidiospores lunate, biapiculate, 15–20 × 3–4.25 µm	***T. junci-acuti***
–	Basidiospores sigmoidal, vermiform, 19–36 × 3–5 µm	***T. vermiferum***
9	Basidiospores not guttulate, flattened at the adaxial side	***T. raphidisporum***
–	Basidiospores guttulate or oily	**10**
10	Cystidia without crystals	**11**
–	Cystidia encrusted with crystals	**13**
11	Basidiospores 18.5–22.5 × 3.2–3.8 µm; cystidia with cylindrical hyphae	***T. naviculisporum***
–	Basidiospores narrower and/or longer; cystidia with dendroidly branching hyphae	**12**
12	Basidiospores 20–29 × 2.2–3 µm; hymenophore pale-orange to greyish-orange	***T. bambusicola***
–	Basidiospores 20–34 × 3–4 µm; hymenophore cream	***T. yunnanense***
13	Crystals polytetrahedral; basidiospores width > 4 μm, 4–4.4 μm	***T. fusiforme***
–	Crystals columnar; basidiospores width < 4 μm, 3–3.5 μm	***T. densum***

## Discussion

In distinguishing *Tubulicium* and *Litschauerella*, several morphological traits are diagnostic, including the chemical reaction, crystal encrustation, and hyphal covering of lyocystidia, as well as basidiospore shape and surface ornamentation. In *Litschauerella*, lyocystidia are typically encrusted with crystals and fibrous hyphae; the inner layer is weakly to strongly dextrinoid, and basidiospores are globose to broadly ellipsoid with fine ornamentation visible in Melzer’s reagent. In contrast, *Tubulicium* possesses lyocystidia, mostly covered by dendroidly branching hyphae with few crystals, a weakly amyloid inner layer, and ellipsoid to vermiform basidiospores that remain smooth in Melzer’s reagent.

Lyocystidia are key morphological features shared by both genera. This structure is relatively uncommon among corticioid fungi but occurs across unrelated phylogenetic lineages: within the *Trechisporales* (in *Dextrinocystis* and *Tubulicium*) and within the *Hymenochaetales* (in *Litschauerella*, *Tubulicrinis* Donk, *Leifia* Ginns, and *Odonticium* Parmasto; Bernicchia and Gorjón 2010; Liu et al. 2019a; He et al. 2020). Recently, Wijesinghe et al. (2025) introduced two monotypic genera, *Grandicium* Y.F. Dai & C.L. Zhao and *Membranohymenium*, in the *Trechisporales*, which were reported to possess lyocystidia-like cystidia. The presence of lyocystidia and lyocystidia-like structures in these genera indicates multiple independent evolutionary origins within *Agaricomycetes*, suggesting morphological convergence. Such repeated evolution may imply adaptive significance, possibly related to basidioma reinforcement, enzymatic secretion, or water retention in wood-decaying environments (Larsson and Ryvarden 2021).

Ecologically, species of *Tubulicium* inhabit a wide range of substrates, including angiosperm wood, bamboo culms, and tree-fern stems (Boidin and Gaignon 1992; Liu et al. 2019b; Ushijima et al. 2019). Environmental sequences related to *Tubulicium* have also been detected from tea (*Camellia sinensis*), orchid roots (*Dendrobium exile*, *Epidendrum firmum*), other angiosperm roots such as *Allanblackia stuhlmannii*, and even soil samples from various regions (Kartzinel et al. 2013; Xing et al. 2017, 2019; de Mesquita et al. 2018, 2020). These findings suggest that some *Tubulicium* species may not be strictly saprotrophic but could form facultative or opportunistic associations with plants, a phenomenon also reported in *Trechispora* (Lin et al. 2025). Species of *Litschauerella* occur on gymnosperm and angiosperm wood and occasionally on fern stems. Sequences closely related to *Litschauerella*, which belong to the same family and lineage, are frequently detected in environmental barcoding studies of soil samples (Taylor et al. 2007, 2014; Eichorst and Kuske 2012; Mueller et al. 2014), suggesting that *Litschauerella* species are primarily saprotrophic and probably widespread in terrestrial habitats.

## Supplementary Material

XML Treatment for Litschauerellaceae

XML Treatment for Litschauerella

XML Treatment for Litschauerella abietis

XML Treatment for Litschauerella clematidis

XML Treatment for Litschauerella gladiola

XML Treatment for Litschauerella hastata

XML Treatment for Tubulicium

XML Treatment for Tubulicium bambusicola

XML Treatment for Tubulicium curvisporum

XML Treatment for Tubulicium densum

XML Treatment for Tubulicium dussii

XML Treatment for Tubulicium fusiforme

XML Treatment for Tubulicium naviculisporum

XML Treatment for Tubulicium raphidisporum

XML Treatment for Tubulicium vermiferum

## Additional information

### Artificial Intelligence (AI) use

The authors accept full responsibility for the content of the manuscript, including the disclosure of any use of AI.

No AI tools were used in the preparation of this manuscript.

### Author ORCIDs

Y.-C. Lin https://orcid.org/0000-0002-0526-5601

C.-C. Chen https://orcid.org/0000-0002-2268-439X

### Data availability

Sanger assembled sequences have been deposited in NCBI. The files of sequence alignments and phylogenetic trees have been deposited at Figshare (https://doi.org/10.6084/m9.figshare.32568702).
